# Nocturnal pulse oxygen saturation dynamics at simulated high altitude: Predictive value for acute mountain sickness in healthy men born pre‐term

**DOI:** 10.1113/EP092418

**Published:** 2025-01-16

**Authors:** Benjamin J. Narang, Giorgio Manferdelli, Grégoire P. Millet, Tadej Debevec

**Affiliations:** ^1^ Department for Automatics Biocybernetics and Robotics, Jožef Stefan Institute Ljubljana Slovenia; ^2^ Faculty of Sport University of Ljubljana Ljubljana Slovenia; ^3^ Institute for Exercise and Environmental Medicine Texas Health Presbyterian Dallas Dallas Texas USA; ^4^ Institute for Sport Sciences University of Lausanne Lausanne Switzerland

**Keywords:** hypoxia, prematurity, pulse oximetry, sleep

## Abstract

The physiological sequelae of pre‐term birth might influence the responses of this population to hypoxia. Moreover, identifying variables associated with development of acute mountain sickness (AMS) remains a key practically significant area of altitude research. We investigated the effects of pre‐term birth on nocturnal oxygen saturation ($S_{pO_{2}}$) dynamics and assessed the predictive potential of nocturnal $S_{pO_{2}}$‐related metrics for morning AMS in 12 healthy adults with gestational age < 32 weeks (pre‐term) and 12 term‐born control participants. Participants spent one night at a simulated altitude of ∼4200 m (normobaric hypoxia; fraction of inspired O_2 _= 0.141), with nocturnal $S_{pO_{2}}$ and heart rate recorded continuously at the fingertip using pulse oximetry and with morning AMS assessed using the Lake Louise scale. Pre‐term and term‐born participants had similar nocturnal mean $S_{pO_{2}}$ (mean ± SD; 77% ± 3% vs. 77% ± 4%; *P* = 0.661), minimum $S_{pO_{2}}$ (median[IQR]; 67[4]% vs. 69[5]%; *P *= 0.223), relative time spent with $S_{pO_{2}}$ < 80% (72% ± 29% vs. 70% ± 27%; *P* = 0.879) and mean heart rate (79 ± 12 vs. 71 ± 7 beats/min; *P* = 0.053). However, the increase in $S_{pO_{2}}$ between the two halves of the night was blunted with prematurity (−0.12% ± 1.51% vs. 1.11% ± 0.78%; *P* = 0.021). Moreover, the cumulative relative desaturation‐based hypoxic ‘load’ was higher with prematurity (32[26]%min/h vs. 7[25]%min/h; *P* = 0.039), underpinned by increased desaturation frequency (69[49] vs. 21[35] counts/h; *P* = 0.009). Mean $S_{pO_{2}}$, minimum $S_{pO_{2}}$, morning $S_{pO_{2}}$ and relative time spent with $S_{pO_{2}}$ < 80% predicted AMS incidence better than a random classifier exclusively in the pre‐term group, with no other variables predictive of AMS in the two groups separately or combined. Overall, pre‐term birth might alter nocturnal $S_{pO_{2}}$ dynamics and influence AMS prediction in severe hypoxia.

## Abstract (highlights)

- **What is the central question of this study?**
  Does pre‐term birth impact nocturnal pulse oxygen saturation dynamics and acute mountain sickness during simulated high‐altitude exposure equivalent to 4200 m?
- **What is the main finding and its importance?**
  Despite no influence of pre‐term birth on composite nocturnal pulse oxygen saturation metrics, an increased desaturation‐induced hypoxic burden was observed in this group. Composite pulse oxygen saturation metrics predicted acute mountain sickness exclusively in pre‐term adults, suggesting a more direct connection between nocturnal hypoxaemia and altitude‐related illness. These findings advance our understanding of prematurity‐related physiology, particularly in relationship to acute prediction of mountain sickness.

## 1 INTRODUCTION

Premature births are characterized by delivery before 37 weeks of gestation (Ohuma et al., 2023). Although survival rates are high, pre‐term birth can lead to lasting physiological effects beyond maturity, even in healthy adults (Crump, 2020). Given that altitude‐induced hypoxia necessitates an integrated adaptation of physiological systems (Wyatt, 2014), the investigation of pre‐term physiology under hypoxic stress holds both mechanistic insight and practical implications (Narang, Manferdelli, Millet, et al., 2022).

Rapid ascents to high altitudes induce acute mountain sickness (AMS), which, in extreme cases, can develop into life‐threatening cerebral oedema (Luks et al., 2017). The prevalence of AMS increases at higher altitudes (Beidleman et al., 2013), occurring in 53% of unacclimatized hikers exposed to 4243 m of terrestrial altitude (Hackett et al., 1976). The identification of factors that reliably predict AMS occurrence remains a fundamental pursuit of high‐altitude research. Several attempts have been made to identify acute resting (Burtscher et al., 2019; Faulhaber et al., 2014; Gatterer et al., 2019; Joyce et al., 2024) or exercise‐related measurements (Cobb et al., 2021; Mairer et al., 2013; Richalet et al., 2012) from which AMS susceptibility might be screened. However, AMS prediction remains challenging, with limited model consistency owing to variations in sample characteristics, environmental exposure protocols and device utilization/accuracy. Nonetheless, an enhanced understanding of how individuals with pre‐existing lung and cardiovascular conditions tolerate altitude exposure is vital for informing guidelines targeting clinical populations.

Premature birth introduces a complex interplay of factors that could influence AMS susceptibility (Debevec et al., 2022). In particular, differences in pulmonary function together with modulated chemoreceptor sensitivity could predispose this population to increased AMS risk (Narang, Manferdelli, Millet, et al., 2022). For example, specific differences in both the magnitude (Bates et al., 2014; Debevec et al., 2019; Manferdelli et al., 2021) and the pattern (Narang et al., 2023) of pulmonary ventilation in response to environmentally induced blood gas challenges have been observed in this cohort. A connection between the awake resting hypoxic ventilatory response and development of AMS seems intuitive, considering the attenuated reduction in pulse oxygen saturation ($S_{pO_{2}}$), but findings in this regard are inconsistent (Chen et al., 2012; Goves et al., 2024; Hohenhaus et al., 1995; Karinen et al., 2010; Milledge et al., 1991; Roach et al., 1998; Wagner et al., 2012). Of interest, however, is the observation that nocturnal hypoxaemia, together with (Nespoulet et al., 2012) or independent from (Eichenberger et al., 1996; Erba et al., 2004; Joyce et al., 2024) hypoventilation, could be associated with AMS incidence. In particular, a recent field study by Joyce et al. (2024) observed high predictive potential for AMS of various $S_{pO_{2}}$‐related metrics recorded overnight during ascent up to 4800 m. Notably, no study has investigated nocturnal $S_{pO_{2}}$ responses at high altitudes in prematurely born individuals, despite their apparent increased risk of sleep‐disordered breathing at sea level (Crump et al., 2019). Moreover, connecting potential differences in nocturnal $S_{pO_{2}}$ induced by pre‐term birth with AMS incidence might prove valuable in understanding the manifestation of altitude‐related illnesses in these individuals.

The primary aim of this study was to compare nocturnal $S_{pO_{2}}$ dynamics between healthy adults born pre‐term and a group of term‐born control participants during a night in severe normobaric hypoxia, equivalent to an altitude of ∼4200 m. Prematurely born participants were hypothesized to demonstrate more frequent and severe desaturations, particularly considering that the risk of sleep‐disordered breathing might also be increased in this cohort. The secondary aim was to identify the predictive potential of these parameters for the classification of AMS+ individuals the following morning. Various nocturnal $S_{pO_{2}}$ metrics were expected to be predictive of AMS incidence regardless of group.

## 2 MATERIALS AND METHODS

### 2.1 Ethical approval

Ethical approval was obtained from the University of Ljubljana Faculty of Sport Ethical Committee (8/2020‐316), and the project was pre‐registered at ClinicalTrials.gov (NCT04739904). All procedures were conducted in accordance with the *Declaration of Helsinki*. Informed consent was obtained from all participants before any testing procedures.

### 2.2 Recruitment and participant characteristics

Participants were eligible if they were healthy, recreationally active men, engaging in structured exercise a minimum of three times per week, aged 18–30 years and free from any known cardiorespiratory diseases. Additionally, pre‐term participants were eligible if they had been born with a gestational age ≤32 weeks and a gestational mass ≤1500 g. Prematurely born individuals fulfilling these recruitment criteria were identified through birth records and personally invited to participate. A convenience sample of well‐matched term‐born individuals was recruited to represent the control group. A total of 29 participants were initially recruited, comprising 15 born pre‐term and 14 born at term. One pre‐term participant withdrew during the study owing to altitude sickness symptoms, which meant that a complete nocturnal $S_{pO_{2}}$ data trace could not be obtained. Additionally, the nocturnal $S_{pO_{2}}$ data trace obtained from four participants was insufficient for analysis. In particular, the signal was lost in the first few hours of the recordings and was not recovered, hence several outcome variables were either not possible to calculate or would be greatly influenced by the nature of the missing data. Overall, a final cohort of 24 participants was included in the analysis, with 12 born prematurely and 12 born at term. Participant characteristics are presented in Table 1. Assessment of pulmonary function was conducted in line with previously reported protocols (Narang et al., 2023), and results are quantified in absolute terms and relative to predicted values based on standardized equations (Quanjer et al., 2012; Stanojevic et al., 2017). The protocols used to assess peak oxygen uptake were also outlined previously (Manferdelli et al., 2023).

**TABLE 1:** Participant characteristics.

Characteristic	Term‐born (*n* = 12)	Pre‐term (*n* = 12)	*P*‐value
Age, years	22 ± 3	21 ± 3	0.180
Body mass index, kg/m^2^	23 ± 2	23 ± 3	0.991
Gestational age, weeks	40 ± 1	29 ± 2	<0.001
Gestational mass, g	3673 ± 454	1071 ± 250	<0.001
${\dot{V}}_{O_{2}\mathrm{peak}}$, mL/kg/min	50.7 ± 7.5	48.2 ± 12.5	0.551
FVC, L	5.8 ± 0.8	5.3 ± 1.0	0.223
FVC, % predicted	97 ± 11	97 ± 10	0.903
FEV_1_, L	4.5 ± 0.9	4.2 ± 0.6	0.306
FEV_1_, % predicted	90 ± 17	90 ± 8	0.937
PEF, L/s	8.8 ± 1.6	8.8 ± 1.4	0.995
DL_CO_, mmol/kPa/min	12.2 ± 1.1	11.0 ± 2.4^a^	0.156
DL_CO_, % predicted	104 ± 12	101 ± 14^a^	0.517
*K*_CO_, coefficient	1.72 ± 0.22	1.68 ± 0.18	0.618
*K*_CO_, % predicted	100 ± 12	96 ± 11	0.431
Alveolar volume, L	7.1 ± 0.7	6.6 ± 1.2^a^	0.164
Alveolar volume, % predicted	104 ± 7	105 ± 10^a^	0.949

*Note*: All values are reported as the mean ± SD. Predicted values for lung function were obtained from up‐to‐date reference equations for spirometry (Quanjer et al., 2012) and lung diffusion capacity (Stanojevic et al., 2017). *P*‐values were obtained from Student's independent two‐tailed *t*‐tests for between‐group comparisons. Significant differences in gestational age and mass are the intuitive result of recruitment criteria.

Abbreviations: DL_CO_, lung diffusion capacity for carbon monoxide; FEV_1_, forced expiratory volume in one second; FVC, forced vital capacity; *K*_CO_, transfer coefficient of the lung for carbon monoxide; PEF, peak expiratory flow rate; ${\dot{V}}_{O_{2}\mathrm{peak}}$, peak oxygen uptake.

^a^ *n* = 11.

### 2.3 Protocol overview

Participants travelled from Ljubljana, Slovenia (295 m) to the Olympic Sports Centre Planica, Slovenia (940 m) between 10.00 and 12.00 h on the day of arrival. They then consumed an ad libitum lunch before 13.00 h and entered the hypoxic facility between 14.00 and 15.00 h. Participants were then confined to the reduced‐oxygen environment until departure the following day. Between 19:15 and 20.00 h, participants consumed an ad libitum dinner. They were then free to relax in the hypoxic room until 21.00 h, at which point they completed the evening questionnaires. After being instrumented for overnight recordings, the lights in the hypoxic bedroom were turned off at 22.00 h. Participants were woken by the researchers at 06.00 h and completed the morning questionnaires at 07.00 h.

### 2.4 Environmental conditions

Environmental conditions within the facility were precisely controlled and remained stable throughout the exposures (barometric pressure = 684 ± 4 mmHg, temperature = 21.3°C ± 1.0°C, relative humidity = 35% ± 3%). Normobaric hypoxia was induced via a vacuum pressure swing adsorption system (VPSA type 85, B‐Cat, Tiel, The Netherlands). Ambient oxygen concentrations were monitored automatically and regulated at 15 min intervals. The fraction of inspired O_2_ was maintained at 0.141 to simulate an altitude of ∼4200 m at a terrestrial altitude of 940 m. The mean partial pressure of inspired oxygen throughout the testing period was 89.9 mmHg. Ambient carbon dioxide levels were monitored throughout the testing period, with the average fraction of inspired CO_2_ corresponding to 0.0016 ± 0.0009 and never exceeding 0.004.

### 2.5 Questionnaires

Acute mountain sickness was assessed using the Lake Louise scale 6 h after hypoxic room entry (21.00 h) and 1 h after waking (07.00 h). The scale requires participants to report scores from zero to three, reflecting the severity of each of the four symptoms: headache, gastrointestinal distress, fatigue and/or weakness and dizziness/light‐headedness (Roach et al., 2018). Participants were classified as AMS+ if their total symptom score was at least three, with a headache score of at least one. Subjective sleep quality was assessed using the Groningen Sleep Quality Scale (GSQS): a questionnaire consisting of 15 statements, for which the response ‘True’ or ‘False’ is given (Meijman et al., 1990). The composite score ranges from 0 to 14, with higher scores indicating lower subjective sleep quality. The GSQS has previously been shown to provide a valid and reliable assessment of perceived sleep quality in a high‐altitude setting (Jafarian et al., 2008).

### 2.6 Nocturnal data recording and processing

A reusable soft pulse‐oximetry sensor (8000SX‐WO, Nonin Medical Inc., Plymouth, MN, USA) was placed on the right index finger and secured using micropore tape. This sensor recorded $S_{pO_{2}}$ and heart rate continuously at 3 Hz from 22.00 to 06.00 h. Data were subsequently processed using MatLab (R2023a, MathWorks, Natick, MA, USA). The central 6 h from each data trace, corresponding to the time frame from 23.00 to 05.00 h, was extracted for analysis. Artefacts within the $S_{pO_{2}}$ measurement traces were identified as absolute values >100% or <30%, and any changes of >4% between consecutive samples. After identification, all measurement artefacts were removed. The $S_{pO_{2}}$ variables calculated from the artefact‐free data trace reflected those investigated in a recent study, in which promising predictive potential of nocturnal $S_{pO_{2}}$ for AMS incidence in terrestrial altitude conditions was established (Joyce et al., 2024). These included the arithmetic mean, the minimum instantaneous value and the proportion of the total 6 h duration for which $S_{pO_{2}}$ was <80% (TST80) (Cross et al., 2015). The difference between the means of the two halves of the recordings was also calculated (∆$S_{pO_{2}}$) (Tannheimer et al., 2017), as was the arithmetic mean of the final hour from the extracted 6 h data trace (from 04.00 to 05.00 h; morning $S_{pO_{2}}$). Mean heart rate was also quantified. Desaturation instances were defined, as previously outlined (Taha et al., 1997), by rate (>0.1%/s), magnitude (≥2% lower than baseline) and re‐saturation pattern (return to within 1% of pre‐event baseline or 3% recovery relative to nadir, whichever occurs first). The total duration from the initial decrease from baseline to re‐saturation was required to be ≥10 and ≤60 s (Taha et al., 1997). The desaturation frequency, or ‘oxygen desaturation index’ (ODI), is reported as a rate per hour. The desaturation duration represents the arithmetic mean of the duration of all desaturation events. The ‘hypoxic burden’ was also calculated from the desaturation events. That is, all areas under the curve (AUCs), relative to their respective pre‐desaturation baseline values, were cumulatively aggregated and expressed in relative terms (%min/h) (Azarbarzin et al., 2018; Joyce et al., 2024).

### 2.7 Statistical analyses

Statistical analyses were conducted using RStudio (R v.4.2.1, R Core Team, Boston, MA, USA). Results for AMS data are reported descriptively. All other data were subjected to inferential statistics. All parameters relating to nocturnal $S_{pO_{2}}$ and heart rate data were assessed for distribution normality using the Shapiro–Wilk test. Between‐group comparisons were subsequently made using Student's independent *t*‐tests or Mann–Whitney *U*‐tests for parametric and non‐parametric data, respectively, and are, in turn, respectively reported as the mean ± SD or as the median[IQR]. Statistical significance was accepted in all cases if *P *< 0.05. Receiver operating characteristic (ROC) analyses were used to indicate the predictive potential of $S_{pO_{2}}$‐ and heart rate‐related parameters for morning AMS incidence. The ROC statistics are reported using their respective AUC values, with 95% confidence intervals computed from 10,000 stratified bootstrap replicates. Predictor variables were considered valuable if the lower limit of the 95% confidence interval of the AUC was >0.5, thus predicting AMS incidence significantly better than a random classifier. In these cases, Youden's index was calculated to identify the optimal balance of sensitivity and specificity across the probability range. These values were then extracted together with their corresponding raw threshold values.

## 3 RESULTS

### 3.1 Questionnaire responses

In the evening, 9 of 12 (75%) term‐born and 7 of 12 (58%) pre‐term participants were AMS+. Upon waking, 5 of 12 (42%) term‐born and 6 of 12 (50%) pre‐term participants were AMS+. Subjective sleep quality was similar between the pre‐term and the term‐born groups (GSQS: 10 ± 3 vs. 9 ± 5; *P* = 0.511).

### 3.2 Nocturnal cardiorespiratory parameters

**FIGURE 1:**
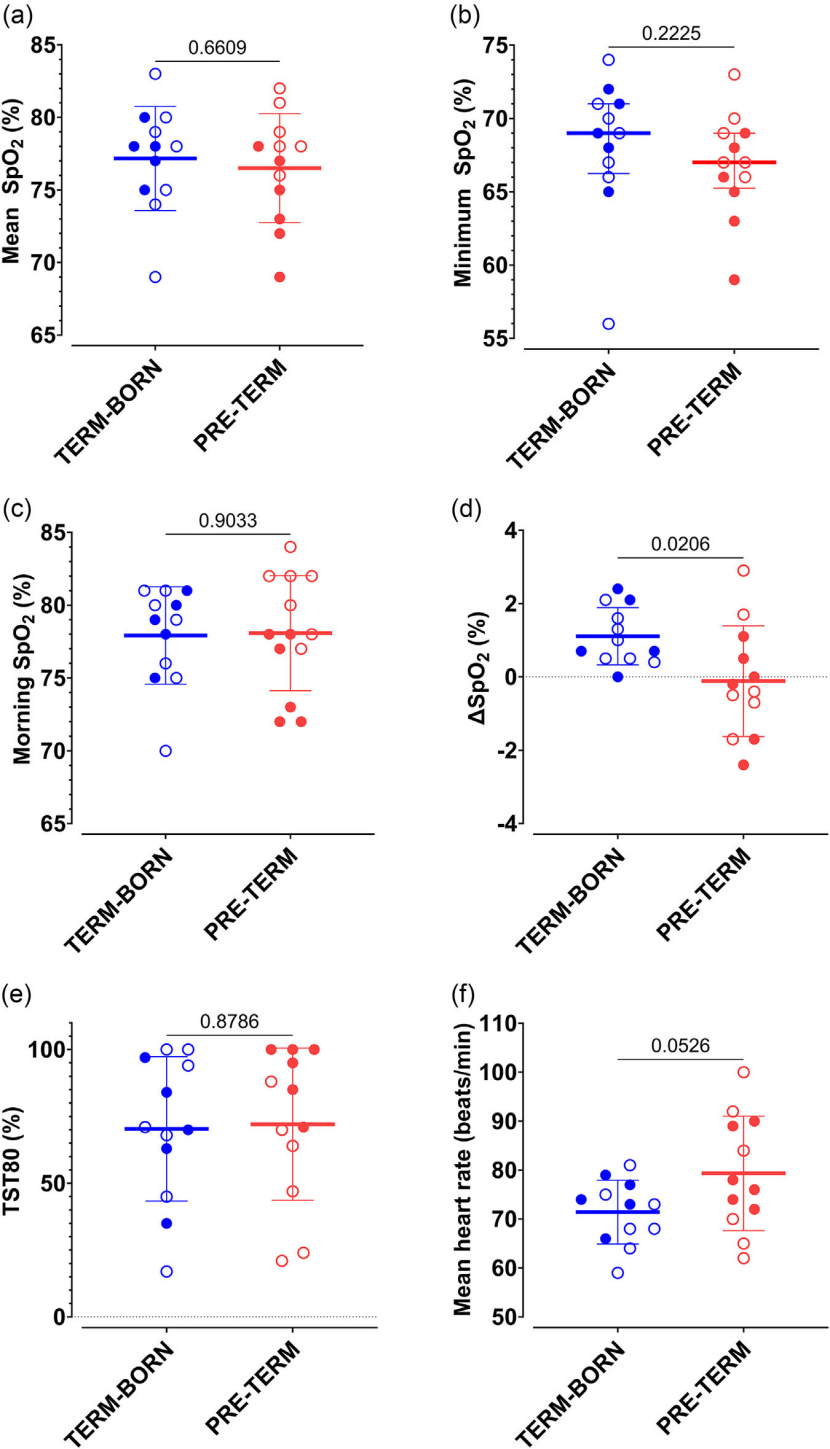
Effects of pre‐term birth on composite nocturnal $S_{pO_{2}}$ and heart rate, including mean $S_{pO_{2}}$ (a), minimum $S_{pO_{2}}$ (b), morning $S_{pO_{2}}$ (c), the increase in $S_{pO_{2}}$ between the two halves of the night (∆$S_{pO_{2}}$; d), the proportion of the night spent with $S_{pO_{2}}$ < 80% (TST80; e) and mean heart rate (f). For normally distributed data, values for individual participant are superimposed over the arithmetic mean, with error bars indicating the SD and the *P*‐value reported as the result of Student's independent two‐tailed *t*‐test (a, d–f). Data that deviated from a normal distribution are represented with individual participant values superimposed over the median, with error bars representing the interquartile range and the *P*‐value reported as the result of a Mann–Whitney *U*‐test (b, c). Term‐born participants are shown in blue and pre‐term participants in red. Filled circles represent participants classified as AMS+ in the morning, whereas open circles depict participants who were not classified as AMS+ upon waking. Abbreviations: AMS, acute mountain sickness; $S_{pO_{2}}$, pulse oxygen saturation.

Nocturnal mean $S_{pO_{2}}$ was similar between the pre‐term and term‐born groups (77% ± 3% vs. 77% ± 4%; *P* = 0.661; Figure 1a), as was minimum $S_{pO_{2}}$ (67[4]% vs. 69[5]%; *P* = 0.223; Figure 1b), morning $S_{pO_{2}}$ (78[7]% vs. 79[6]%; *P* = 0.903; Figure 1c) and TST80 (72%% ± 29% vs. 70% ± 27%; *P* = 0.879; Figure 1e). However, ∆$S_{pO_{2}}$ was lower in the pre‐term participants (−0.12% ± 1.51%) than in their term‐born counterparts (1.11% ± 0.78%; *P* = 0.021; Figure 1d). Mean heart rate did not differ significantly between the pre‐term and the term‐born groups (79 ± 12 vs. 71 ± 7 beats/min; *P* = 0.053; Figure 1f).

**FIGURE 2:**
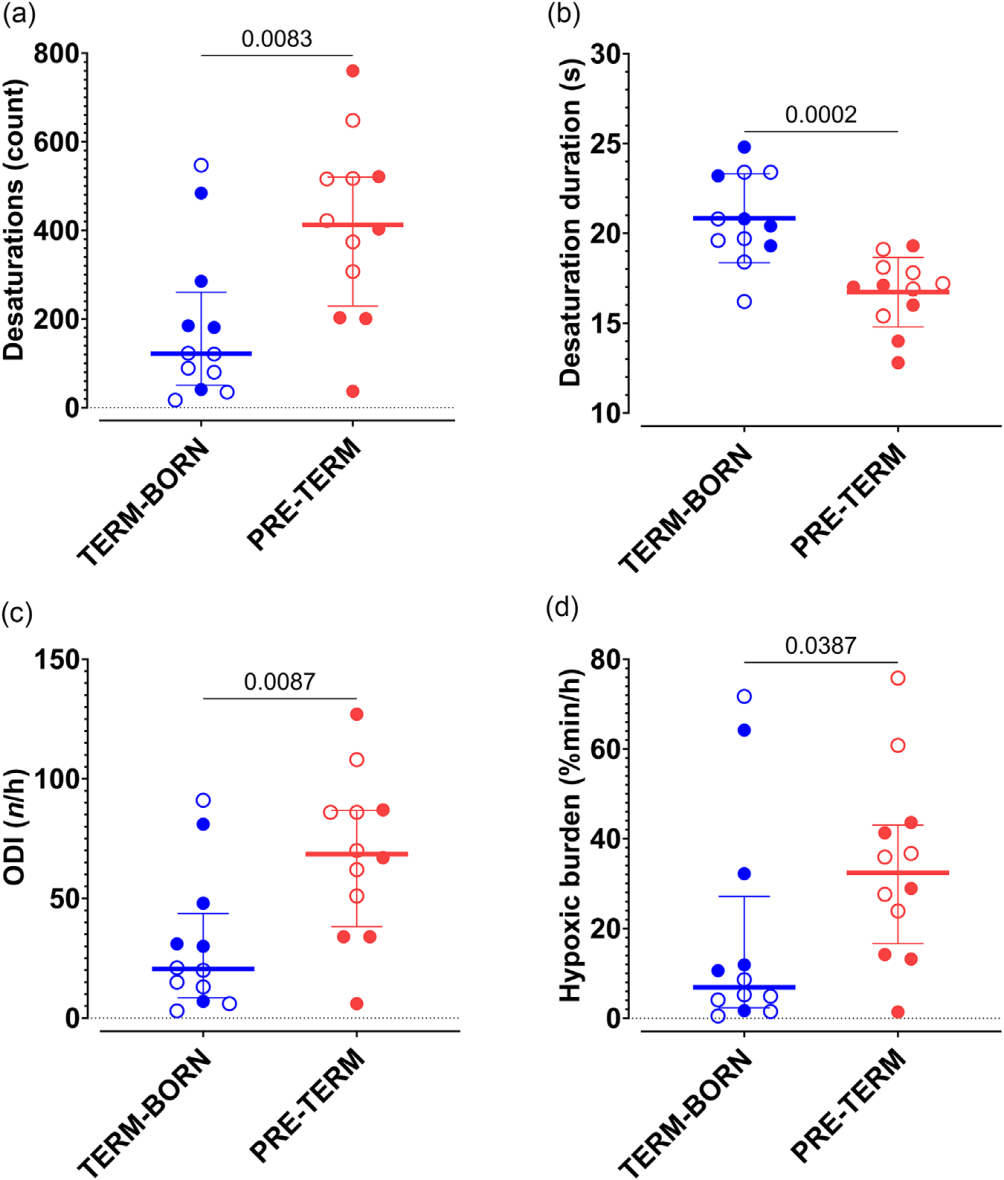
Effects of pre‐term birth on desaturation‐related metrics, including the total number of desaturations (a), the mean desaturation duration (b), the ODI (c) and the total relative hypoxic burden calculated from all desaturation events (d). For normally distributed data, values for individual participants are superimposed over the arithmetic mean, with error bars indicating the SD and the *P*‐value reported as the result of Student's independent two‐tailed *t*‐test. Data that deviated from a normal distribution are represented with values for individual participants superimposed over the median, with error bars representing the interquartile range and the *P*‐value reported as the result of a Mann–Whitney *U*‐test. Term‐born participants are shown in blue and pre‐term participants in red. Filled circles represent participants classified as AMS+ in the morning, whereas open circles depict participants who were not classified as AMS+ upon waking. Abbreviations: AMS, acute mountain sickness; ODI, oxygen desaturation index.

Pre‐term adults experienced more total desaturations (413[291] vs. 122[209] counts; *P* = 0.008; Figure 2a), hence a greater ODI (69[49] vs. 21[35] counts/h; *P* = 0.009; Figure 2b). The desaturations experienced by the pre‐term group were, however, shorter in average duration (17 ± 2 vs. 21 ± 2 s; *P* < 0.001; Figure 2c). Overall, pre‐term participants experienced a significantly greater desaturation‐induced hypoxic burden through the night (32[26]%min/h vs. 7[25]%min/h; *P* = 0.039; Figure 2d).

### 3.3 Prediction of AMS

Table 2 contains the results of the ROC analyses for the potential of nocturnal cardiorespiratory parameters to predict morning AMS incidence. Data are reported for all participants together and for the two groups separately. Of note, mean $S_{pO_{2}}$, minimum $S_{pO_{2}}$, morning $S_{pO_{2}}$ and TST80 predicted morning AMS incidence better than a random classifier exclusively in the prematurely born individuals. No other parameters were able to classify AMS+ participants significantly better than a random classifier in the two groups separately or combined. Threshold values from the raw data, together with their corresponding sensitivity and specificity, are reported for the pre‐term group in the four variables that demonstrated high predictability.

**TABLE 2:** Results of the receiver operating characteristic analyses for the potential of various nocturnal parameters to predict correctly those participants with acute mountain sickness in the morning.

Predictor variable	All participants	Term‐born only	Pre‐term only
Mean $S_{pO_{2}}$, %	0.706 (0.483–0.902)	0.457 (0.143–0.829)	0.889 (0.639–1.000)^a^
Minimum $S_{pO_{2}}$, %	0.626 (0.381–0.843)	0.543 (0.200–0.871)	0.806 (0.514–1.000)^a^
Morning $S_{pO_{2}}$, %	0.708 (0.477–0.892)	0.514 (0.171–0.857)	0.933 (0.667–1.000)^a^
TST80, %	0.678 (0.448–0.888)	0.571 (0.229–0.886)	0.944 (0.778–1.000)^a^
∆$S_{pO_{2}}$, %	0.545 (0.308–0.769)	0.429 (0.086–0.829)	0.472 (0.139–0.833)
Mean heart rate, beats/min	0.650 (0.413–0.874)	0.686 (0.343–1.000)	0.444 (0.083–0.833)
Desaturations, count	0.476 (0.245–0.706)	0.743 (0.400–1.000)	0.639 (0.278–0.972)
ODI, count/h			
Desaturation duration, s	0.469 (0.231–0.720)	0.629 (0.286–0.914)	0.694 (0.333–1.000)
Hypoxic burden, %min/h	0.483 (0.245–0.720)	0.743 (0.400–1.000)	0.722 (0.389–0.972)

Predictor variable	Threshold value	Sensitivity (%)	Specificity (%)
Mean $S_{pO_{2}}$, %	77.3	83	83
Minimum $S_{pO_{2}}$, %	66.5	67	83
Morning $S_{pO_{2}}$, %	76.9	83	83
TST80, %	78.1	100	83

*Note*: Reported values are the areas under the receiver operating characteristic curves, with the 95% confidence interval ranges reported in parentheses. Values for ‘Desaturations, count’ and ‘ODI, count/h’ are identical, because the latter is derived directly from the former by dividing each value by a constant (6 h). Predictor variables that performed significantly better than a random classifier were assessed further to determine the optimal threshold value, based on an equivalently weighted balance between sensitivity and specificity, the values of which are also reported. Note that these additional analyses were carried out only using the data from the pre‐term group (*n* = 12).

Abbreviations: AMS, acute mountain sickness; ODI, oxygen desaturation index (number of desaturations per hour); $S_{pO_{2}}$ pulse oxygen saturation; TST80, the proportion of nocturnal recording duration spent with $S_{pO_{2}}$ < 80%.

^a^ Predictor variable performs significantly better than a random classifier (95% confidence intervals do not cross 0.500).

## 4 DISCUSSION

In the present study, we investigated nocturnal hypoxaemia and $S_{pO_{2}}$ dynamics in healthy adults born pre‐term compared with term‐born control participants during exposure to normobaric hypoxia equivalent to ∼4200 m altitude. Composite metrics did not differ between the two groups, whereas overnight $S_{pO_{2}}$ recovery was lower in the pre‐term participants, and desaturations induced a greater relative hypoxic burden in this group. The composite $S_{pO_{2}}$ variables predicted AMS occurrences well in the pre‐term participants, whereas desaturation‐related metrics did not. No nocturnal $S_{pO_{2}}$ variable reliably classified AMS incidence in the term‐born group or when the groups were assessed collectively.

The comparable nocturnal mean $S_{pO_{2}}$, minimum $S_{pO_{2}}$, morning $S_{pO_{2}}$ and TST80 between pre‐term and term‐born participants suggest that overall absolute altitude‐induced nocturnal hypoxaemia was similar between the two groups. Contemporary evidence suggests that healthy adults born prematurely nearer the turn of the millennium display more positive outcomes with regard to both lung function (Bårdsen et al., 2022) and chemosensitivity to hypoxia (Narang et al., 2023; Narang, Manferdelli, Kepic, et al., 2022) than their counterparts born up to 30 years earlier (Bates et al., 2014). The observation, therefore, that the participants in the present study maintained similar absolute nocturnal $S_{pO_{2}}$ relative to their term‐born counterparts is perhaps unsurprising and constitutes a generally promising observation from a clinical perspective. These findings lend further support to the critical consideration of the dynamicity of experimental research connected with prematurity. Technological advancements to improve neonatal care processes have not only increased pre‐term survival rates but also seem to have improved the lasting phenotype of individuals born at a certain gestational age (Hubbard et al., 2023).

Despite no differences in overall composite metrics of nocturnal $S_{pO_{2}}$, a notable difference was observed in ∆$S_{pO_{2}}$, indicating a reduced capacity for nocturnal $S_{pO_{2}}$ recovery in pre‐term individuals. $S_{pO_{2}}$ exhibits a diurnal rhythm, whereby peak $S_{pO_{2}}$ tends to occur close to midday, and the lowest $S_{pO_{2}}$ values are recorded around midnight (Shapiro et al., 2023). In this respect, nocturnal increases in $S_{pO_{2}}$ can be considered the natural result of a diurnal fluctuation, which, in an altitude‐related context with drastically reduced $S_{pO_{2}}$ values, might be particularly important with regard to physiological function. Indeed, nocturnal ∆$S_{pO_{2}}$ was quantified in a previous field study at high altitude, with the authors proposing that the metric could be reflective of the degree of acclimatization of an individual (Tannheimer et al., 2017). In the present study, 11 of the 12 term‐born participants had a positive ∆$S_{pO_{2}}$, with the exception demonstrating a value of only −0.02%. Conversely, 8 of the 12 pre‐term adults presented with a decrease in $S_{pO_{2}}$ throughout the night. Considering that the morning $S_{pO_{2}}$ data were similar between the two groups in this study, it is possible that earlier adaptation might have occurred in the prematurely born participants. Comparisons of $S_{pO_{2}}$ early in the nocturnal recording period seemed confounded by between‐individual variability in sleep/wake status, hence this remains only a speculative point. Importantly, however, the apparently diminished ability to recover $S_{pO_{2}}$ levels in pre‐term individuals did not appear to contribute to an increased AMS susceptibility in this study.

Nocturnal desaturations are commonly observed in patients with chronic respiratory diseases (Lacasse et al., 2011), and desaturation frequency has been independently associated with cardiovascular disease (Wang et al., 2022). In the present study, the pre‐term group experienced a greater desaturation frequency throughout the night, perhaps reflecting underlying differences in ventilatory control mechanisms. For example, we previously showed that pre‐term adults exhibit a breathing pattern with more pronounced fluctuations when breathing a hypoxic hypercapnic gas mixture during an awake, acute (6 min) exposure (Narang et al., 2023). This suggests clearer rhythmicity in the pattern of pulmonary ventilation in conditions that might be triggered by the apnoea/hypopnoea episodes associated with nocturnal periodic breathing. The more frequent desaturations observed in the pre‐term participants of the present study therefore support the notion that a subconscious underlying pattern of rhythmic breathing, consisting of regular, cyclic fluctuations in resting ventilation, is present in prematurely born adults. In turn, the integration of central and peripheral chemoreceptors in sensing and responding to hypercapnic and hypoxic episodes remains a worthwhile pursuit of prematurity‐related research. Furthermore, although the desaturations were, on average, shorter in duration, the overall relative hypoxic burden was greater in the pre‐term group. These findings concur with a population‐based cross‐sectional analysis that found a 40% higher relative risk of sleep‐disordered breathing with pre‐term birth, even after various perinatal and maternal factors were considered (Crump et al., 2019). The risk of sleep‐disordered breathing at sea level was not assessed in the present study, hence specific ventilatory responses to blood gas challenges and nocturnal periodic breathing risk both remain potential candidates for the desaturation‐related differences observed. Crucially, these differences did not translate to a higher incidence of AMS in prematurely born individuals. As a result, these findings provide more of a basis for further mechanistic research, rather than necessarily helping to predict clinical outcomes or to contribute to altitude‐related travel guidance. In relation to the need for further mechanistic research, no studies have yet investigated the specific effects of intermittent hypoxia in this population. Investigating responses such as long‐term facilitation in relation to intermittent hypoxic exposures could provide valuable insights into the mechanisms underpinning the observed patterns of nocturnal desaturations, as these may even confer health benefits according to a recent review (Burtscher et al., 2024). We therefore consider this to be a worthwhile avenue of continued investigation, particularly in relation to the long‐term effects of premature birth and potential for related interventions.

In the present study, composite metrics of absolute nocturnal hypoxaemia were similar between groups, but AMS prediction using these variables was successful exclusively in the pre‐term group. Conversely, relative $S_{pO_{2}}$ measures differed between the pre‐term and term‐born group, but did not predict AMS in either group. Taken together, these observations suggest that pre‐term birth does not influence absolute nocturnal $S_{pO_{2}}$, but the connection between absolute nocturnal $S_{pO_{2}}$ and AMS is more consistent with prematurity. Mechanistic differences that might underlie these observations must therefore exist at the connection between absolute $S_{pO_{2}}$ levels and the development of AMS. In line with this reasoning, we propose three speculative mechanisms underpinning a closer association between hypoxaemia and AMS with prematurity. First, considering that the cardinal symptom for AMS classification is a headache (Roach et al., 2018) and that cerebral effects underpin the development of AMS (Wilson et al., 2009), it might be that pre‐term birth modulates cerebral responses to hypoxaemia. Indeed, the altitude‐induced increase in cerebral blood flow and cerebrovascular conductance was recently shown to be impaired in prematurely born healthy adults (Manferdelli, Narang, Bourdillon, Giardini, et al., 2023). With a reduced arterial oxygenation, increased oxygen extraction by the brain would be required to maintain cerebral oxygen delivery. Second, prematurely born individuals maintain left ventricular functional limitations beyond maturity (Huckstep et al., 2018), which could impair oxygen delivery to metabolically active tissue related to AMS symptoms including the brain, gastrointestinal system and vestibular system. A lower oxygen delivery to these tissues might limit their metabolic processes, increasing the perception of symptoms such as dizziness and digestive discomfort. Third, baroreflex sensitivity has been shown to be blunted in hypoxia in prematurely born adults (Manferdelli et al., 2024). In line with the potential limitations induced by right ventricular impairments, the impaired blood pressure regulation might hinder the appropriate redistribution of blood flow to target tissues such as those outlined above, while simultaneously contributing to cardiovascular instability. In any case, these mechanisms remain highly speculative, and further research using larger study cohorts is needed to validate the observation that absolute nocturnal hypoxaemia predicts AMS outcomes more accurately in pre‐term individuals. Then, specific high‐altitude nocturnal studies that aim to elucidate underlying mechanisms would be warranted.

Much of the existing high‐altitude literature offers insights into acute physiological assessments that may, or may not, contribute to a predictive model of AMS susceptibility (Burtscher et al., 2019; Cobb et al., 2021; Faulhaber et al., 2014; Gatterer et al., 2019; Joyce et al., 2024; Mairer et al., 2013; Richalet et al., 2012). The present study contributes to this literature base, with prolonged $S_{pO_{2}}$ recordings in the relatively ‘vulnerable’ context of nocturnal physiology. The use of a high‐altitude simulation allowed for close scientific control of the experimental environment and the opportunity to readily induce AMS, owing to the instant transition to hypoxia. However, this equally represents an ecological validity limitation, considering that normobaric hypoxia has been proposed to manifest differently from a physiological perspective in comparison to the hypobaric hypoxia induced by high‐altitude travel (Millet & Debevec, 2020). A key limitation in the present study is the lack of polysomnography data, which precluded quantification of sleep/wake time and prevented objective measurement of sleep quality. The lack of polysomnographic variables might have also limited our ability to predict AMS more accurately, as these variables could have provided further insight. That said, using pulse oximetry alone is highly relevant and practically applicable, because these devices are commercially available and commonly used in the field. It is also worth considering that a larger sample size might be necessary to substantiate the observations of the present study. In particular, ROC analysis is strengthened when data are divided into a model training set for logistic regression and a test set for evaluation, to minimize data overfitting to the model. Moreover, a larger number of participants would improve the precision of the identified thresholds, in addition to the resolution of the sensitivity and specificity estimates. The participants recruited for the present study were a relatively homogeneous cohort of prematurely born and term‐born participants, according to the prespecified inclusion criteria. We therefore assume that undiagnosed disease incidence is distributed randomly across the two cohorts and unlikely to confound the results of the study. For example, patent foramen ovale is associated with AMS (West & Tobin, 2024). However, to our knowledge, evidence is yet to robustly demonstrate an increased prevalence of patent foramen ovale in the prematurely born adult population. Moreover, the association between patent foramen ovale and AMS is lost under normobaric hypoxia (DiMarco et al., 2022), in conditions similar to those used in this study. Lastly, a few considerations warrant discussion with regard to the male‐only cohort tested in the present study. The incidence of AMS has been proposed to be higher in females (Richalet et al., 2012), possibly due to a reduced hypoxic ventilatory response and more pronounced hypoxaemia in normobaric hypoxic conditions (Camacho‐Cardenosa et al., 2022). Sex‐specific recommendations for women venturing to high altitudes are emerging as a result of these observations (Burtscher et al., 2023). Conversely, the female sex has been associated with lower rates of pre‐term birth (Peelen et al., 2016) and more favourable outcomes with regard to prematurity‐related morbidity (Peacock et al., 2012). Ultimately, it is likely that sex differences would moderate the variables measured in this study, and follow‐up investigations are necessary to develop sex‐specific guidelines.

## 5 CONCLUSION

Overall, the present data indicate that prematurely born male adults exhibit a greater relative hypoxic burden, underpinned by more frequent desaturations and impaired overnight $S_{pO_{2}}$ recovery, during a high‐altitude simulation at 4200 m. Interestingly, desaturation‐related metrics of nocturnal $S_{pO_{2}}$ did not predict AMS incidence in either group of participants in this study. On the contrary, although composite metrics of $S_{pO_{2}}$ (mean, minimum, morning and TST80) do not appear to differ between the two populations, they do predict AMS incidence in prematurely born adults specifically. Further research seems warranted to elucidate underlying mechanisms that might result in these associations, in addition to translating the findings to inform interventions aimed at mitigating AMS risk in this population.

## AUTHOR CONTRIBUTIONS

Conceived and designed research: Benjamin J. Narang, Giorgio Manferdelli, Grégoire P. Millet and Tadej Debevec Performed experiments: Benjamin J. Narang and Giorgio Manferdelli. Analysed data: Benjamin J. Narang. Interpreted results of experiments: Benjamin J. Narang. Prepared figures: Benjamin J. Narang. Drafted manuscript: Benjamin J. Narang. Edited and revised manuscript: Benjamin J. Narang, Grégoire P. Millet and Tadej Debevec All authors approved the final version of the manuscript and agree to be accountable for all aspects of the work in ensuring that questions related to the accuracy or integrity of any part of the work are appropriately investigated and resolved. All persons designated as authors qualify for authorship, and all those who qualify for authorship are listed.

## CONFLICT OF INTEREST

None declared.

## Data Availability

The anonymized data collected are available as open data via the Zenodo online data repository: https://doi.org/10.5281/zenodo.11505046. The corresponding author may be contacted for any questions regarding the data or any other content relating to the manuscript.
